# Impact of acute mental stress on ankle blood pressure in young healthy men: a pilot study

**DOI:** 10.1186/s13104-022-06160-7

**Published:** 2022-07-30

**Authors:** Daisuke Kume, Masato Nishiwaki, Norio Hotta, Hiroshi Endoh

**Affiliations:** 1 Faculty of Information Science and Technology, Osaka Institute of Technology, 1-79-1 Kitayama, Hirakata, Osaka 573-0171 Japan; 2 Department of Health, Sports and Welfare, Okinawa University, 555 Kokuba, Naha, Okinawa 902-8521 Japan; 3 Faculty of Engineering, Osaka Institute of Technology, 5-16-1 Omiya, Asahi-ku, Osaka, 535-8585 Japan; 4 Department of Lifelong Sports and Health Sciences, Chubu University, 1200 Matsumoto-cho, Kasugai, Aichi 487-8501 Japan; 5 Department of Health and Physical Education, University of the Ryukyus, 1 Senbaru, Nishihara, Okinawa 903-0213 Japan

**Keywords:** Brachial blood pressure, Ankle blood pressure, Pressor response, Mental arithmetic, Cardiovascular diseases

## Abstract

**Objective:**

Acute mental stress (MS) increases arm blood pressure (BP); however, it remains unclear whether a stress-induced pressor response is also observed in other vessels. This study aimed to examine the impact of acute MS on ankle BP. Fifty-six young, healthy men aged 19–24 years were divided into the MS (n = 29) and control (CON) (n = 27) groups; each group performed 5-min MS (mental arithmetic) or CON tasks. Systolic and diastolic BPs (SBP and DBP, respectively) of both the brachial and posterior tibial arteries were simultaneously measured at the baseline and 5 and 30 min after the task.

**Results:**

In the MS group, brachial BP measures significantly increased (*P* < 0.05) until 30 min after the task; ankle BP measures were also significantly (*P* < 0.05) elevated during this time. In the CON group, no significant changes were found in brachial BP measures or ankle SBP, whereas a significant increase (*P* < 0.05) in ankle DBP was observed 30 min after the task. Our findings indicate that both brachial and ankle BP exhibit a sustained elevation after acute MS, suggesting a systemic pressor response by stress exposure. The measurement of ankle BP in addition to arm BP may be important to assess the stress response.

**Trial Registration:**

UMIN Clinical Trials Registry UMIN000047796 Registered on: 20th May 2022.

## Introduction

An increase in blood pressure (BP) is known to be a typical human cardiovascular response to acute mental stress (MS). Arm BP increases during acute MS; this pressor response reportedly persists for a long time after the cessation of stress exposure [1–4], but not in all cases [5, 6]. Acute MS in the laboratory is considered as a proxy of stressful exposures during daily life [7]. Indeed, an exaggerated increase in arm BP during acute MS and a delayed BP recovery after stress exposure are associated with an increased risk of future cardiovascular disease (CVD) [8]. Therefore, assessing BP responses to acute MS has potential clinical implications.

Importantly, the impact of acute MS on BP in other vessels besides arm BP has not yet been investigated. Recently, the importance of assessing lower limb arterial health has been proposed in detecting general CVD risk [9]. A reference for normal ankle systolic BP (SBP) has previously been provided for clinical and research studies [10], and a chronic increase in ankle SBP is an independent predictor of CVD [11, 12]. If ankle BP increases with acute MS, similar to arm BP, chronic repetition of episodic ankle pressor responses due to stressful exposures during daily life may contribute to elevating the basal BP levels of the same site. However, the changes in ankle BP associated with acute MS are currently unclear. Therefore, the present pilot study aimed to investigate the impact of acute MS on ankle BP.

## Main text

### Methods

#### Participants

A total of 56 young healthy male college students (age range, 19–24 years) from Okinawa University participated in the study. Due to the COVID-19 pandemic, which made the recruitment of middle-aged and elderly adults difficult, only young people were included, and the study had a relatively small sample size. The participants were divided into the MS (n = 29; age, 20.6 ± 0.2 years; height, 169.1 ± 0.9 cm; body weight, 63.8 ± 1.2 kg; body mass index, 22.3 ± 0.4 kg/m^2^ [means ± SE]) and CON groups (n = 27; age, 20.4 ± 0.2 years; height, 167.6 ± 0.8 cm; body weight, 64.2 ± 1.6 kg; body mass index, 22.8 ± 0.4 kg/m^2^). None of the participants were smokers or took any medications during the study period. The purpose, experimental procedure, and risks associated with the study were fully explained to all participants, and they provided written informed consent. The study was approved by the Ethics Committee of Okinawa University (#2018–03) and conducted in accordance with the guidelines of the Declaration of Helsinki.

#### Experimental procedures

All experiments were conducted in a quiet, air-conditioned room (24ºC–26ºC) at Okinawa University. All participants were asked to refrain from performing strenuous exercise, consuming alcohol (≥ 24 h) and caffeine (≥ 12 h), and eating (≥ 3 h) before the experiments.

**Fig. 1:**
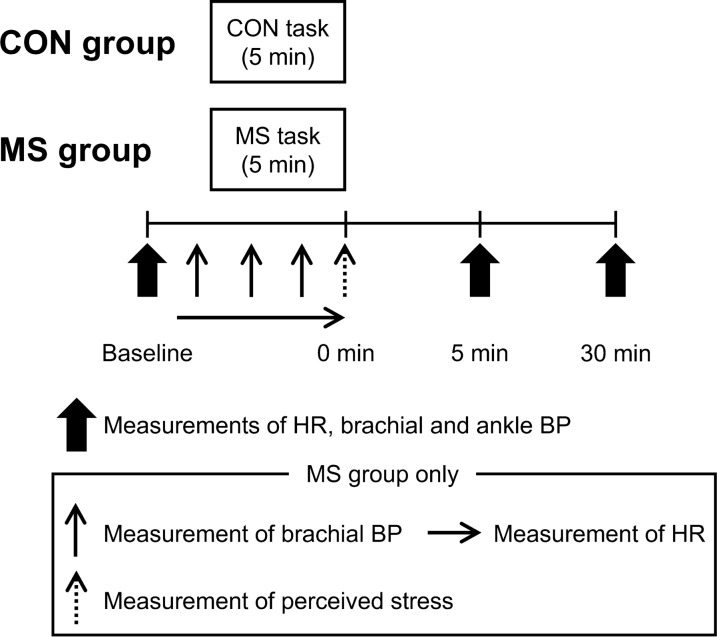
Experimental protocol. CON, control; MS, mental stress; BP, blood pressure; HR, heart rate

A schematic representation of the experimental protocol is shown in Fig. 1. All participants were positioned supine throughout the experimental session. After resting in the supine position for at least 15 min, baseline measures of hemodynamic variables were obtained. Subsequently, the MS and CON groups performed either a 5-min MS or a CON task (detailed below); a post-task phase was set to 30 min.

We used mental arithmetic as the MS task according to previous studies [6, 13]. To elaborate, each participant was asked to serially subtract 13 from a 3-digit number (close to 1,000) as quickly and accurately as possible within 5 min. During the task, the participants were intentionally frustrated by being asked to perform the calculation faster and by being corrected immediately if wrong answers were provided. A metronome was played loudly for additional distraction. When the number was < 13 (the answer was not allowed to go below 0), the participants restarted the task using the original 3-digit number. On the other hand, in the CON task, the participants were instructed to slowly count upward, starting from 1, for 5 min, as previously described [6, 14], without any annoying instructions or metronome noises.

#### Measurements

In addition to the baseline measures, heart rate (HR), SBP, and diastolic BP (BP) of both the brachial and posterior tibial arteries were measured at 5 min and 30 min after the task using a vascular testing system (VaSera VS-1500AN; Fukuda Denshi, Tokyo, Japan). For the measurements, BP cuffs were wrapped on both upper arms and ankles, and electrocardiogram electrodes were placed on both wrists.

To assess the stress responses, we measured the brachial SBP and DBP using an automated sphygmomanometer (Tango + ; SunTech Medical Instruments, Morrisville, NC, USA) before and during the MS task. During the task, the measurement was conducted twice (approximately 2 and 4 min after starting the task), and the average value was calculated. HR was also continuously measured using a three-lead electrocardiogram (413; Intercross, Tokyo, Japan). HR data were recorded simultaneously with the BP measurements and averaged. Further, immediately after the task, the participants were asked to rate their perceived stress during the task using a standard five-point scale of 0 (not stressful), 1 (somewhat stressful), 2 (stressful), 3 (very stressful), and 4 (very, very stressful) [15], which was selected based on previous studies [16, 17]. On the other hand, these assessments were not conducted in the CON task because no marked changes in hemodynamic variables and perceived stress rating in response to this task have been previously confirmed [6, 14].

#### Statistical analysis

Data are expressed as means ± SE. Hemodynamic variables before and during the MS task were compared using a paired student’s t-test. Two-way (time × group) repeated-measures analysis of variance (ANOVA) with Bonferroni-corrected post-hoc testing was performed on the post-task changes in hemodynamic variables. The significance was considered at a *P-*value < 0.05. Statistical analyses were conducted using SPSS version 28.0 software (IBM SPSS Japan, Tokyo, Japan).

## Results

In the MS group, marked increases in HR and brachial BP in response to the task were observed (Table 1), and the mean perceived stress level was 2.8 ± 0.2 on a five-point scale.

**Table 1:** The response of hemodynamic variables to the task in the MS group

	Before the task	During the task
HR (beats/min)	58 ± 1	74 ± 2*
Brachial SBP (mmHg)	112 ± 1	128 ± 2*
Brachial DBP (mmHg)	60 ± 1	77 ± 2*

Data are expressed as means ± SE

*MS* mental stress, *HR* heart rate, *SBP* systolic blood pressure *DBP* diastolic blood pressure

^*^P < 0.05 vs. before the MS task

The post-task changes in HR as well as the brachial and ankle BP measurements are illustrated in Table 2. The mean baseline ankle SBP in the CON and MS groups were 132 and 130 mmHg, respectively, which was within the normal range for young people with normal arm SBP [10]. As the main results, brachial SBP and DBP significantly increased until at 30 min after the task in the MS group. Similarly, ankle SBP and DBP were significantly elevated during this time in the same group. In the CON group, no significant change was found in brachial BP measures and ankle SBP across the measurement timepoints, whereas a significant increase in ankle DBP was observed at 30 min after the task.

**Table 2:** Post-task changes in the heart rate and brachial and ankle blood pressure measures in both groups

	Group	Baseline	5 min	30 min	ANOVA
HR (beats/min)	CON	57 ± 2	57 ± 1	56 ± 1	Interaction: *P* = 0.026
	MS	57 ± 1	59 ± 1*	56 ± 1	
Brachial SBP (mmHg)	CON	120 ± 2	118 ± 2*	120 ± 2	Interaction: *P* < 0.001
	MS	118 ± 1	121 ± 1*	120 ± 1*	
Brachial DBP (mmHg)	CON	69 ± 1	69 ± 1	70 ± 1	Time: *P* = 0.010; Group: *P* = 0.576
	MS	68 ± 1	71 ± 1*	71 ± 1*	Interaction: *P* = 0.077
Ankle SBP (mmHg)	CON	132 ± 2	132 ± 2	132 ± 2	Interaction: *P* < 0.001
	MS	130 ± 2	136 ± 2*	137 ± 2*	
Ankle DBP (mmHg)	CON	68 ± 1	68 ± 1	70 ± 1*	Interaction: *P* = 0.049
	MS	67 ± 1	70 ± 1*	72 ± 1*	

Data are expressed as means ± SE

*HR* heart rate, *SBP* systolic blood pressure, *DBP* diastolic blood pressure, *CON* control, *MS* mental stress, *ANOVA* analysis of variance

^*^P < 0.05 vs. baseline

## Discussion

Our study demonstrates for the first time that not only brachial BP but also ankle BP exhibit a sustained elevation after acute MS.

Elevations in HR and BP are well known as typical cardiovascular responses during acute MS [1–6, 13, 15–17]. In this study, HR and brachial BP were strikingly increased during the task in the MS group; the perceived stress rating was also comparable with that reported in previous studies [16, 17]. Therefore, we believe that MS was intentionally elicited in the MS group.

In this study, the increased brachial BP induced by acute MS persisted for 30 min post-stress, which is congruent with previous reports demonstrating long-lasting arm BP elevations after acute MS [1–4]. On the other hand, other studies have reported the absence of a sustained pressor response post-stress [5, 6]. The reason for the inconsistency among the studies may be due to the differences in the participant characteristics, task types or durations, or BP measurement method.

In addition to the above brachial pressor response, we found an increase in ankle BP after acute MS, which was sustained for 30 min. Acute MS is known to substantially increases the circulating levels of catecholamines [18, 19], which would affect various tissues via blood circulation. In addition, robust elevation in muscle sympathetic nerve activity (MSNA) has been observed after acute MS [15, 16], which can be observed in both upper and lower limbs [16], suggesting the presence of a systemic effect. Both norepinephrine infusion and elevated MSNA increase vascular resistance of vessels in the limbs [20–22]. Moreover, it has recently been suggested that delayed BP recovery after acute MS occurs, in part, via a neurogenic vascular mechanism mediated by α_1_-adrenergic receptors [23]. Considered together, we speculate that the observed brachial and ankle pressor response after acute MS is likely to be attributed to the increased sympathetic vasoconstrictor tone at a systemic level. Direct evidence supporting our assertion is not available, and further investigations, therefore, are warranted. On the other hand, we observed an increase in ankle DBP at 30 min after the task in the CON group. Such vascular response seems to be caused by the basal vascular tone to maintain the peripheral blood flow, and the observed increase in ankle DBP in the MS group might also be partially due to the same mechanism.

It has been reported that a marked arm pressor response to acute MS is linked to CVD [8]. In this study, we provide the first evidence demonstrating that acute MS results in a long-lasting elevation in ankle BP. Therefore, repeated exposure to increased ankle BP due to stressful exposures during daily life activities may cause a chronic elevation of ankle BP, which is identified as an independent predictor of CVD [11, 12]. Although this aspect is largely speculative at this time, we consider that the measurement of ankle BP in addition to arm BP can be used as an important assessment of stress response, and this would further expand our understanding of the association between human cardiovascular response to acute MS and CVD.

In conclusion, the present study demonstrates that acute MS results in a sustained elevation in BP in both upper (i.e., the brachium) and lower (i.e., the ankle) limbs vessels in young, healthy men, suggesting a stress-induced systemic pressor response.

## Limitations

This study has the following limitations. First, only young men were included. This was due to the difficulty in recruiting other populations, and we sought to examine young male individuals in this pilot study. Further studies in other populations, such as women, older individuals, and hypertensive patients, are needed because inter-population differences in vascular response to acute MS have been reported previously [24–26]. Second, ankle BP measurements were not taken during acute MS; this should be addressed in future studies to formulate the assessment of stress response using ankle BP.

## Data Availability

The data that support the findings of this study are available from the corresponding author upon reasonable request.

## Abbreviations

BP: Blood pressure
CON: Control
CVD: Cardiovascular disease
DBP: Diastolic blood pressure
HR: Heart rate
MS: Mental stress
SBP: Systolic blood pressure
